# Airfoil Selection Procedure, Wind Tunnel Experimentation and Implementation of 6DOF Modeling on a Flying Wing Micro Aerial Vehicle

**DOI:** 10.3390/mi11060553

**Published:** 2020-05-30

**Authors:** Taimur Ali Shams, Syed Irtiza Ali Shah, Ali Javed, Syed Hossein Raza Hamdani

**Affiliations:** 1 Department of Aerospace Engineering, College of Aeronautical Engineering, National University of Sciences and Technology, Islamabad 44000, Pakistan; irtiza_shah@gatech.edu (S.I.A.S.); ali.javed@cae.nust.edu.pk (A.J.); 2 Department of Aeronautics and Astronautics, Institute of Space Technology, Islamabad 44000, Pakistan; hossein.hamdani@ist.edu.pk

**Keywords:** flying wing micro aerial vehicle, wind tunnel experimentation, flight tests, selection of reflexed airfoil, 6-DOF modeling

## Abstract

Airfoil selection procedure, wind tunnel testing and an implementation of 6-DOF model on flying wing micro aerial vehicle (FWMAV) has been proposed in this research. The selection procedure of airfoil has been developed by considering parameters related to aerodynamic efficiency and flight stability. Airfoil aerodynamic parameters have been calculated using a potential flow solver for ten candidate airfoils. Eppler-387 proved to be the most efficient reflexed airfoil and therefore was selected for fabrication and further flight testing of vehicle. Elevon control surfaces have been designed and evaluated for longitudinal and lateral control. The vehicle was fabricated using hot wire machine with EPP styrofoam of density 50 Kg/$m^{3}$. Static aerodynamic coefficients were evaluated using wind tunnel tests conducted at cruise velocity of 20 m/s for varying angles of attack. Rate derivatives and elevon control derivatives have also been calculated. Equations of motion for FWMAV have been written in a body axis system yielding a 6-DOF model. It was found during flight tests that vehicle conducted coordinated turns with no appreciable adverse yaw. Since FWMAV was not designed with a vertical stabilizer and rudder control surface, directional stability was therefore augmented through winglets and high wing leading edge sweep. Major problems encountered during flight tests were related to left rolling tendency. The left roll tendency was found inherent to clockwise rotating propeller as ‘P’ factor, gyroscopic precession, torque effect and spiraling slipstream. To achieve successful flights, many actions were required including removal of excessive play from elevon control rods, active actuation of control surfaces, enhanced launch speed during take off, and increased throttle control during initial phase of flight. FWMAV flew many successful stable flights in which intended mission profile was accomplished, thereby validating the proposed airfoil selection procedure, modeling technique and proposed design.

## 1. Introduction

Interest in small creatures flying at low speeds has increased for the last three decades. Research and development organization (commonly known as RAND) conducted a workshop for Advanced Research Projects Agency (ARPA) on “Future Technology Driven Revolutions in Military Operations” in 1992 which resulted in the birth of Micro Aerial Vehicles [1]. Two of the early micro aerial vehicles were “MITE” with its various variants and “Black Widow” [2,3]. Since then, many successful micro aerial vehicles were developed and flight tested. Initially designed micro aerial vehicles were fixed wing in flying wing configurations having low aspect ratio wing for lifting characteristics. These were battery powered (lithium ion or nickel cadmium) and propeller driven with endurance up to 30 min [4,5,6,7,8,9]. In early stages of MAV development, extensive studies were conducted in low Reynolds number aerodynamics regime in which research focused on laminar boundary layer separation, transition to turbulent boundary layer and reattachment to form laminar separation bubble [10]. Low speed wind tunnel experimentation were also conducted to validate theoretical results along with wake measurements for authentic drag predictions [11]. In the same era, researchers conducted research in slow moving low aspect ratio wings. This posed a special problem of three-dimensional flows, where wing tip vortices captured most of the wing area and degraded lift with enhanced drag. Therefore, degradation of lift was not only caused by laminar separation bubble bursting at high angles of attack, but also from large value of induced drag. Therefore, lower value of lift to drag ratio made small aspect ratio wings difficult to fly at slow speeds. In recent past with the advancements of experimental facilities, an extensive experimental research work was conducted on low aspect ratio wings at slow speeds by [12,13,14,15,16,17,18,19,20]. They concluded that low aspect ratio wings at low Reynolds number posed many problems relating to aerodynamic efficiency. Nonlinearity of lift curve, inefficiency of wing planform shapes, and aerodynamic characteristics dependency on aspect ratios were prominent problems. It was found that inverse Zimmerman and rectangular wings were prudent choice of planform. Furthermore, span efficiency value in case of airfoil section was not found to be in conformance with commonly used values in aeronautics literature.

Apart from aerodynamic inefficiency of low aspect wings, flight dynamic considerations were difficult for correct estimation during design phase. Design of MAV is an intricate process where considerable understanding of aerodynamic coefficients as well as rate and acceleration derivatives are essential [21]. Since these are low mass moment of inertia vehicles, they are therefore susceptible to oscillations due to inputs coming from either operator or atmospheric gusts, resulting in flight instabilities. In order to design and analyze a flight dynamic model of MAV, their aerodynamic derivatives are required to be obtained and analyzed. Conventionally, there are three ways in which these aerodynamic derivatives can be estimated. Wind tunnel experiments, computational methods (potential solver or Computational Fluid Dynamic solution), and empirical relations provided in university text books [22]. Since these vehicles are very small compared to man flown flying machines, their geometrical, performance and stability data are not available in open literature. Additionally, the conventional design process as proposed by Daniel Raymer cannot be applied in the design of micro aerial vehicle because of a lack of statistical data concerning these vehicles [23]. This research paper discusses the design of micro aerial vehicles in flying wing configuration where airfoil selection procedure is proposed and explained in detail. Wind tunnel testing of micro aerial vehicles is conducted and aerodynamic coefficients are computed for later use in 6-DOF modeling. Elevon control authority is calculated using potential flow solver ‘XFLRv5’ where control derivatives are calculated with changing elevon deflections. This research paper is organized into five sections. Section 2 deals with the design aspect of flying wing MAV in flying wing configuration. In Section 3, a description of wind tunnel and calibration methods followed by wind tunnel experiments are explained and deliberated upon. Section 4 discusses methodology to calculate elevon control authority, and, in the last section, 6 Degrees of Freedom equations of motion have been formulated.

## 2. Full Scale Design of FWMAV

**Figure 1:**
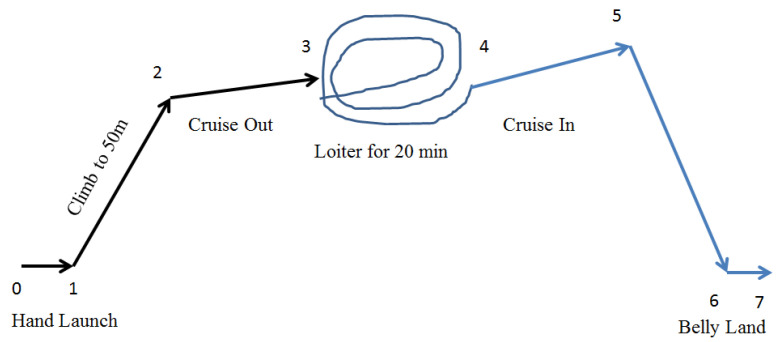
Mission profile consisting of hand launch, steady climb, steady cruise and steady descend mission segments.

Design of any vehicle starts with identification of its purpose and Request for Proposal (RFP). In this research, a Flying Wing Micro Aerial Vehicle (FWMAV) is designed for RSTA missions which are reconnaissance, surveillance and target acquisition. In terms of flying machine, performance can be defined as a measure of its ability to carry out a specified task. Performance of FWMAV is related to stable flight for maximum endurance so that RSTA missions can be performed with ease. The aim is to design a FWMAV with 30 min of endurance, having gross take off weight of 200 grams and capability of camera transmission to ground station from an altitude of 100 m. FWMAV is generally hand launched and belly landed. Mission profile constitutes steady state flight regimes of hand launch, climb, cruise, descend and belly land (refer Figure 1). Since until 100 m of an altitude, air density does not vary by more than 5%, it is assumed that steady state flight conditions prevail [22].

### 2.1. Airfoil Selection

The available wind tunnel test section cross sectional area was 2feet×3feet; therefore, a restriction was imposed on the span of FWMAV to avoid solid blockage and wake blockage errors during wind tunnel experimentation. Hence, vehicle size was selected so that tunnel blockage did not exceed 5%. Model span was kept within 20% of test section width so that wing tip vortices could grow easily and 3D flow conditions are recorded. In addition to model size, structural similarity and strength of support mechanism were ensured at maximum test conditions including maximum angle of attack at highest free stream velocity. Accuracy of a tested model is of prime importance if an accurate prediction of aerodynamic and stability parameters are desired. The model was fabricated using EPP foam with density 30 g/L using CNC machine. EPP (Expanded Polypropylene) has been intensively used in the RC hobby model planes industry and well known as its lightweight physical property, excellent resistance to impact and abrasion [24]. Since the tested model was flying wing configuration, particular importance was therefore given to airfoil shape selection for which a new procedure is proposed in this research paper.

**Figure 2:**
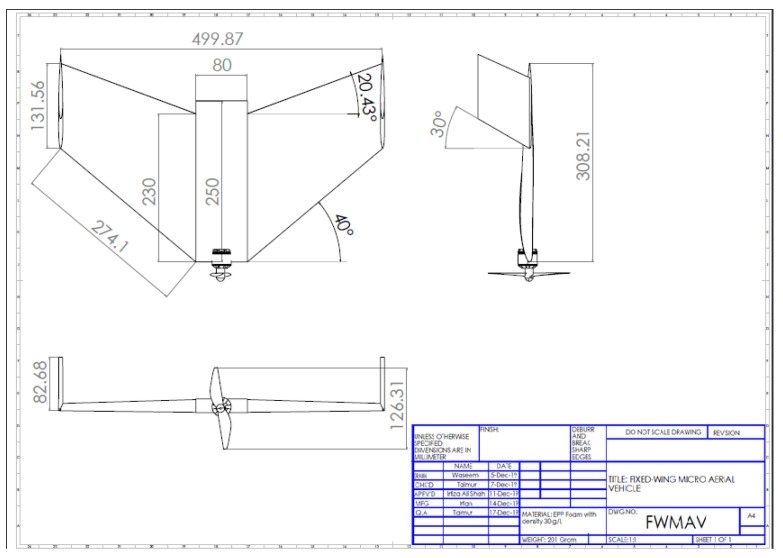
Detailed design features of FWMAV under consideration. All measurements are in milimeters.

For trim flight, pitching moment coefficient at zero angle of attack, $C_{m0}$, must be positive, whereas, for longitudinal stability, aerodynamic center must be aft of CG. For these two requirements, positively cambered airfoil cannot be used for flying wing configuration because this has negative $C_{m0}$. Symmetric airfoil produces zero pitching moment and negatively cambered airfoil produces a positive pitching moment. Negatively cambered airfoil does not fulfill lift requirement; therefore, a negatively cambered airfoil having positive $C_{m0}$ cannot be used in a flying wing configuration airplane. The solution is use of positive cambered airfoil with negative camber at trailing edge to neutralize pitching moment at zero angle of attack. This type of an airfoil is called reflexed airfoil and is considered to be best suited for flying wing configurations. Reflexed airfoils fulfill both the requirements of lift and pitching moment. Selection of best reflexed airfoil for flying wing micro aerial vehicle is discussed in detail in the next sub section. In order to reduce induced drag and to augment lateral stability, carefully designed winglets were installed on the wing tips. A detailed design of model is shown in Figure 2.

### 2.2. Proposed Airfoil Analysis

Selection of reflexed airfoil is a cumbersome process; therefore, a strategy was developed which compared ten reflexed airfoils for selection of an optimum airfoil for flying wing micro aerial vehicles. Reflexed airfoils were selected as candidate airfoils since these nullify the requirement of a horizontal stabilizer for flying wing configuration. These airfoils were computationally analyzed using XFLRv5 software which is a potential flow solver designed by Drela [25].

XFLR5 is an analysis tool for airfoils through XFOIL, wings and planes operating at low Reynolds Numbers. It includes XFoil’s direct and inverse analysis capabilities. Wing design and analysis is based on the Lifting Line Theory, Vortex Lattice Method, and 3D Panel Method. VLM places one horseshoe or ring vortex on each elementary panel which ensures one tangential flow condition on each elementary panel. It estimates aerodynamic derivatives well, except when wing trailing vortices intersect elevator and fin. Since FWMAV was not designed with a horizontal tail or vertical tail, VLM therefore gave authentic results. XFLR treats Navier–Stokes equations as Euler’s equations where viscosity effects are nullified. A further condition of irrotational flow makes Euler’s equation, a potential flow equation. Time independent, incompressible potential flow gives Laplace’s equation (∇Φ=0), which XFLR solves numerically. It is important to highlight that, in XFLRv5, 2D viscous results are imported through XFOIL where 2D Laplace’s equation is solved for velocity field. This velocity field is used as an input in XFOIL to solve a boundary layer problem through simultaneous IBL(Interactive Boundary Layer) methodology [26]. XFOIL evaluates total viscous drag in the wake of airfoil using a Squire–Young formula. The Squire–Young formula is widely used to calculate profile drag in two-dimensional airfoil analysis [27]. Correct estimation of viscous drag and correct location of transition are known limitations of XFLRv5.

**Figure 3:**
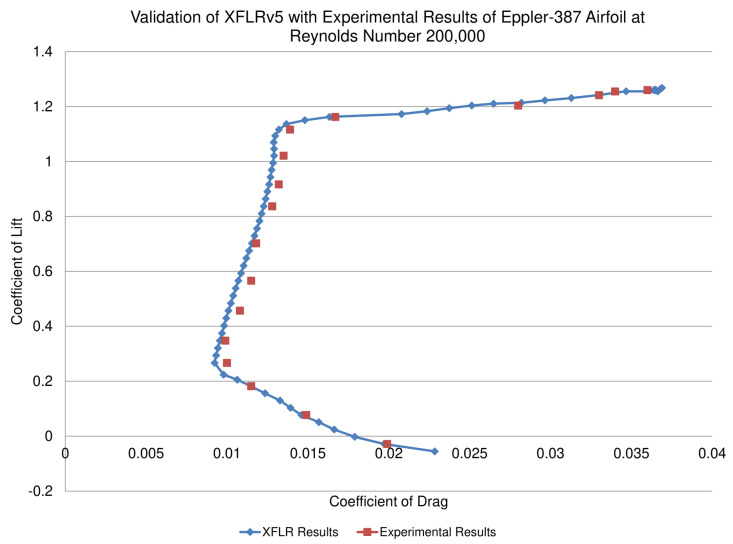
Comparison of numerical results obtained from XFLRv5 with experimental results obtained from [29] for E387 airfoil.

Validation of XFLR software for low Reynolds number airfoils with CFD solver was carried out by Morgado [28]. He compared XFOIL with SST kω turbulence and k−kl−ω transition models to predict airfoil aerodynamic performance parameter at 200,000 Reynolds number. He concluded that XFOIL code gave overall best prediction results. He further declared that XFOIL can predict airfoil performance data with greater accuracy as compared to CFD turbulence models with boundary layer transition detection capability. To further strengthen validation of XFLR, Eppler-387 was analyzed in XFLRv5 software and drag polar as obtained by Maughmer and Selig experimentally was compared with XFLR result [29,30]. Since there is very little difference between the two, XFLRv5 results are therefore dependable and trustworthy. Results shown in Figure 3 show that all predicted values are in good agreement with experimental values. XFLRv5 is found to accurately predict the low drag regions with slightly higher values of coefficient of lift.

### 2.3. Criterion for Selection of Airfoil

The strategy for selection of suitable airfoil considered aerodynamics and stability considerations. Since various parameters define aerodynamic efficiency of airfoil in varying flight regimes, therefore all concerned parameters are computed and proposed parameters τ (airfoil efficiency parameter) and η (airfoil performance parameter) were defined and calculated:

(1) $$\tau =\tau (\frac{C_{l}}{C_{dmax}}+\frac{{C_{l}}^{3/2}}{C_{dmax}}+\frac{{C_{l}}^{1/2}}{C_{dmax}}+C_{l0}+C_{lmax}+\alpha_{stall}+C_{l\alpha }+C_{d0}+C_{m\alpha }+C_{m0})$$

Parameters comprised of aerodynamic efficiency and stability considerations were included in τ function as proposed in Equation (1). Variants of lift to drag ratio and maximum lift coefficient with corresponding angle of attack were placed under aerodynamic efficiency, whereas parameters like $C_{l\alpha }$, $C_{m0}$, and $C_{m\alpha }$ were placed under stability consideration. Maximum endurance and minimum power for propeller driven vehicles correspond to $C_{l}^{3/2}/C_{dmax}$, whereas minimum glide angle and maximum range correspond to $C_{l}/C_{dmax}$. ${C_{l}}^{1/2}/C_{dmax}$ was included in the proposed equation because of its relation with Carson’s speed for optimum cruise speed [31].

For stability parameters, z-force and x-force derivative with respect to forward velocity $Z_{u}$ and $X_{u}$ were considered. $Z_{u}$ is defined as $Z_{u}=\frac{-(C_{lu}+2C_{l0})\cdot QS}{mu_{0}}$[32]. $C_{lu}$ in incompressible subsonic flow regime is considered to be zero, whereas large values of $C_{l0}$ are desired for lift considerations at zero angle of attack, primarily for cruise flight. However, low values of $Z_{u}$ are desired to get lower natural frequency in Phugoid mode $\omega_{np}=\sqrt{-Z_{u}g/u_{0}}$[32]. The added advantage of reduced natural frequency is enhanced damping ratio for phugoid mode $\xi_{p}=-X_{u}/2\omega_{np}$ as discussed in flight stability and automatic control by Robert C Nelson [32]. Understanding the importance of lower values of $C_{l0}$ for better handling qualities in longitudinal mode, it is still desired that airfoil with large value of $C_{l0}$ is selected primarily for aerodynamic considerations and not for stability considerations. The main purpose of airfoil is to generate lift, whereas stability can be handled by placing CG location near the nose of FWMAV. For $X_{u}$, a lower value is desired through selection of airfoil that has a lower value of $C_{d0}$. $C_{du}$ for subsonic incompressible flow regime is zero; therefore, $X_{u}$ value is governed by $C_{d0}$. It is highlighted that $C_{du}$ and $C_{d0}$ play their part in determination of thrust required and a much less contribution goes into stability consideration; however, airfoils with lower values of $C_{d0}$ are desired.

In the similar way, a high damping ratio requirement in short period mode requires high values of lift curve slope $C_{l\alpha }$ through $\xi_{sp}$, where $\xi_{sp}=\frac{-(M_{q}+M_{\dot{\alpha }}+Z_{w})}{2\omega_{n}}$ and $Z_{w}=-\left(C_{l\alpha }+C_{d0}\right)\left(QS/mu_{0}\right)$[32]. Likewise, large values of $M_{q}$ and $M_{\dot{\alpha }}$ are desired for high damping ratio in short period mode. It is, however, stated that since FWMAV was not designed with a separate horizontal tail, rate of change of angle of attack derivatives will therefore be zero. An unstable, yet aerodynamically efficient vehicle is of no use, until a stability augmentation system has been designed for it. If a vehicle has to fly without such arrangement, then requirement of stability supersedes aerodynamic requirements. Aerodynamic requirements are restricted to provide lift to uplift weight of vehicles.

### 2.4. Methodology of Experiments Conducted and Airfoil Selection

A non-dimensional parameter τ was a conceived which consisted of factors from aerodynamic efficiency and stability considerations. τ was made a function of factors as mentioned in Equation (1). In the proposed equation of τ, the aerodynamic efficiency factors were added first and then multiplied with stability efficiency factors, enforcing that stability requirements were considered to be more stringent in any vehicle design. These factors would reject any airfoil which was aerodynamically efficient, but unstable in pitch axis of motion. With this strategy, ten reflexed airfoils were selected for analysis purposes which were E184, E186, E387, FX69H083, NACA M5, NACA M6, S5010, S5020, MH60, and HS-522. It is stated that one airfoil was computationally analyzed from $-5^{\circ }$ to $+18^{\circ }$ angle of attack for 10 aerodynamic coefficients using XFLRv5 software. Therefore, 10 experiments were conducted for each of 10 airfoils. Each experiment generated 23 data points against each angle of attack. Hence, in total, 100 experiments were conducted which generated 2300 values for 10 airfoils. These 2300 values of aerodynamic coefficients were then plotted against angle of attack and maximum value of each of them was extracted which was then included in Table 1 of a manuscript. As a sample calculation, S-5010 airfoil experiments were conducted at Reynolds number of 200,000 and all values are shown in Appendix C. Similar data were generated for remaining nine airfoils. Using Appendix C data, Table 1 values were generated where only maximum value was mentioned for each airfoil. Maximum value in a single column has been shown with bold font to identify maximum value of that column. This value was then used to calculate τ values using Equation (2) and shown in Table 2:

(2) $$\tau =\frac{C_{l}/C_{dmax}}{60.90}+\frac{{C_{l}}^{3/2}/C_{dmax}}{65.98}+\frac{{C_{l}}^{1/2}/C_{dmax}}{57.27}+\frac{C_{lmax}}{1.35}+\frac{\alpha_{stall}}{13.5}+\frac{0.013}{C_{d0}}+\frac{C_{l}0}{0.42}\times (\frac{C_{l\alpha }}{0.1028}+\frac{C_{m\alpha }}{0.0058}+\frac{\left|-0.0087\right|}{C_{m0}}).$$

For the calculation of τ, non-dimensional forms were formed by dividing every parameter with its maximum value. For example, $\frac{C_{l}}{C_{dmax}}$ is divided by a maximum value of $\frac{C_{l}}{C_{dmax}}$ calculated for all airfoils, which is 60.90, therefore $\frac{C_{l}}{C_{dmax}}$ was non-dimensionalized with 60.90. Likewise, all other parameters are divided by their maximum values in order to non-dimensionalize them. Maximum value in each column has been shown with bold font. It is mentioned however, that minimum values of $C_{d0}$ and $C_{m0}$ are desired, therefore they appear in numerator rather than in denominator in Equation (2). Minimum values of $C_{d0}$ and $C_{m0}$ are also shown in bold font for easy identification.

(3) $$\eta =0.2\cdot \frac{C_{l}}{C_{dmax}}+0.4\cdot \frac{{C_{l}}^{3/2}}{C_{dmax}}+0.4\cdot \frac{{C_{l}}^{1/2}}{C_{dmax}}$$

**Figure 4:**
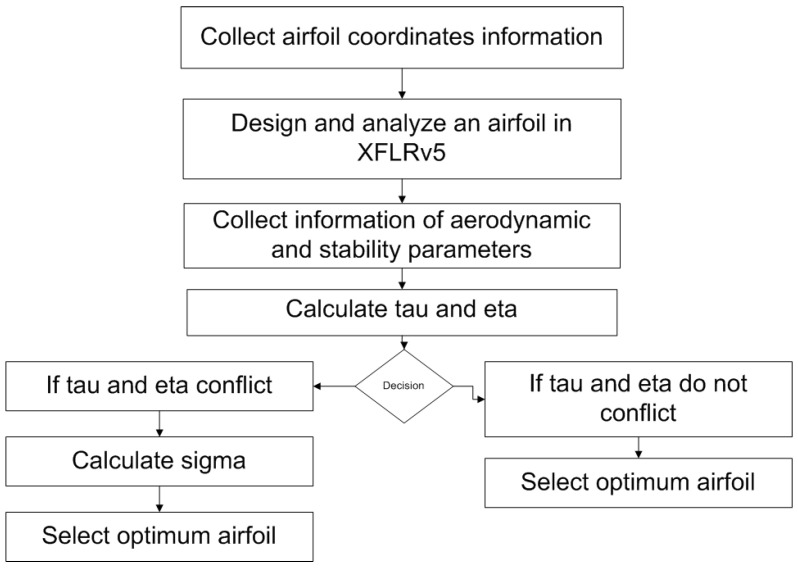
Flow chart of the proposed reflexed airfoil selection procedure for FWMAV.

Airfoil with maximum value of τ is desired for selection of an optimum airfoil. It was found that Eppler-387 has the maximum value of τ, therefore it was short listed as a candidate airfoil. As far as mission profile is concerned, the aerodynamic performance is related to ${C_{l}}^{1/2}/C_{dmax}$, ${C_{l}}^{3/2}/C_{dmax}$ and $C_{l}/C_{dmax}$ refer ([31]). Therefore, another parameter known as performance efficiency parameter,η, was formulated and calculated. The weight fractions were decided on the basis of time fraction of each segment in mission profile. A weight fraction of 0.2 with $C_{l}/C_{dmax}$ was decided on the basis of 6 min of gliding flight in a total of 30 min of flight. A 12-minute cruise and 12 min of flight at minimum power setting correspond to 0.4 weight fraction each. η was calculated as shown in Equation (3). Once η was calculated for airfoils, Eppler-387 gave a maximum of 61.084 (refer to Table 2). The selection criterion for Eppler-387 was made on the highest value of τ and η. However, in the case when one airfoil can not be selected by securing maximum value, an average of τ and η denoted by Σ is to be calculated. For instance, if τ is highest for one airfoil and η is highest for another airfoil, then the highest value of Σ will decide about the selection of a reflexed airfoil. A flow chart of the proposed airfoil selection procedure is shown in Figure 4.

### 2.5. Other Design Features

Leading edge sweep of $40^{\circ }$, trailing edge sweep of $20.43^{\circ }$ and dihedral of $2^{\circ }$ were included in wing design due to lateral stability. The values of leading edge sweep and dihedral angle are considered to be optimum by past researchers [33,34]. Roll stability could be enhanced by various ways including wing position on fuselage, dihedral angle, sweep angle and positioning of vertical stabilizer. Since FWMAV was not designed with vertical stabilizer due to complex control problems, therefore lateral stability was augmented by wing leading edge sweep and wing dihedral angle. In a swept back wing, windward wing has an effective decrease in sweep angle; therefore, it generates more lift as compared to trailing wing. This decrease in sweep from windward wing tries to counter-rotate the wing and hence, enhance lateral stability through differential lift. Apart from lateral stability, wing leading edge sweep augments lift through leading edge vortices [35]. Despite the fact that FWMAV is a slow moving flying wing, a high wing sweep was designed knowingly that wing sweeps are used for high speed aircraft for increase in critical Mach number. Swept wings also augment directional stability by enforcing more air flow over leading wing during side slip. Geometric features of designed micro aerial vehicle are shown in Table 3.

## 3. Wind Tunnel Equipment and Calibrations

Estimation of stability and performance parameters by analytical means is a difficult task which often results in inaccurate formulations. This results in values which are not dependable for analysis purposes. In order to estimate parameters more accurately, wind tunnel tests are to be carried out [36]. More dependable results will be for full scale models where tests are conducted at Reynolds number of actual flight conditions. In that case, both dynamic similarity parameters, which are Reynolds number and Mach number, are matched with actual flight conditions. Therefore, if flow features and dynamics are matched, then wind tunnel results are dependable for flight dynamic analysis. In addition to similarity problems, errors are likely to arise from calibration of equipment and instrumentation used for measurements.

In this research, wind tunnel tests were conducted in 2feet×3feet rectangular cross sectional wind tunnel held with College of Aeronautical Engineering, National University of Sciences and Technology, Pakistan. This wind tunnel is of closed circuit, closed test section, horizontal type, wind tunnel which is capable of producing speeds up to 110 m/s at atmospheric pressure. The corresponding Reynolds number range is from $2.09\times 10^{5}$ to $2.3\times 10^{6}$ (based on 0.3048 m characteristics length of an object) while dynamic pressure range is from 1.84 psfa to 156 psfa. The test section is 6 feet long with rectangular cross sectional shape suitable for pyramidal or sting balance. A wind tunnel consists of motor drive, flow conditioning, contraction nozzle and control console. The drive system of wind tunnel consists of a 150 HP electric motor designed for ambient temperature of $40^{\circ }$ Celsius. The motor drives fan in CCW direction which is equipped with variable pitch propeller to permit continuous pitch change. The fan speed is up to 1500 RPM and propellers are made of special wood to withstand aerodynamic loads during high speed rotation. Flow conditioning primarily consists of diffuser, stilling chamber and contraction region. Turbulence attenuation is achieved by honey comb grid while screen is in the stilling chamber. Subsonic fixed contour converging nozzle accelerates the flow from stilling chamber to the rectangular test section. Since converging nozzle is fixed, desired test section speed is achieved by varying propeller pitch through electric controller. The control console allows remote control of test section velocity and model’s pitch and yaw attitude. Inside the test section, pitch attitude of aircraft model can be altered within $\pm 30^{\circ }$, whereas yaw attitude can be altered within $\pm 90^{\circ }$. Force and moments reading are reflected on a computer screen through three-axis pyramidal strain gauge balance.

### 3.1. Velocity Calibration

Tunnel is instrumented to indicate total temperature and total pressure in stilling chamber and static pressure in the test section. From pressure differential, tunnel speed is indicated on inclined tube manometer, which is known as air meter. Air meter manometer indicates tunnel speed in miles per hour. Liquid inside manometer tube is methyl alcohol with specific gravity of 0.812 at standard atmospheric pressure and temperature. The velocity shown by air meter is calibrated by pitot-static tube mounted inside test section. During velocity calibration of wind tunnel, it was found that velocity through pitot-static probe indicates a maximum of 1.776% velocity under estimation by air meter in low speed range (Table 4). In reduction of test data of Pitot-static tube, allowance was made for tip error and stem error, but compressibility effects were ignored because of low air velocities. During testing, total temperature of the circulating air in the tunnel was observed to increase by $5^{\circ }$ to $7^{\circ }$ due to viscous dissipation. Table 4 shows error in velocities between air meter and pitot-static probe placed inside the test section. The speed range is from 5.64 m/s to 22.35 m/s. These velocities are corresponding to 10 mph and 50 mph, respectively.

### 3.2. Angle of Attack Calibration

**Figure 5:**
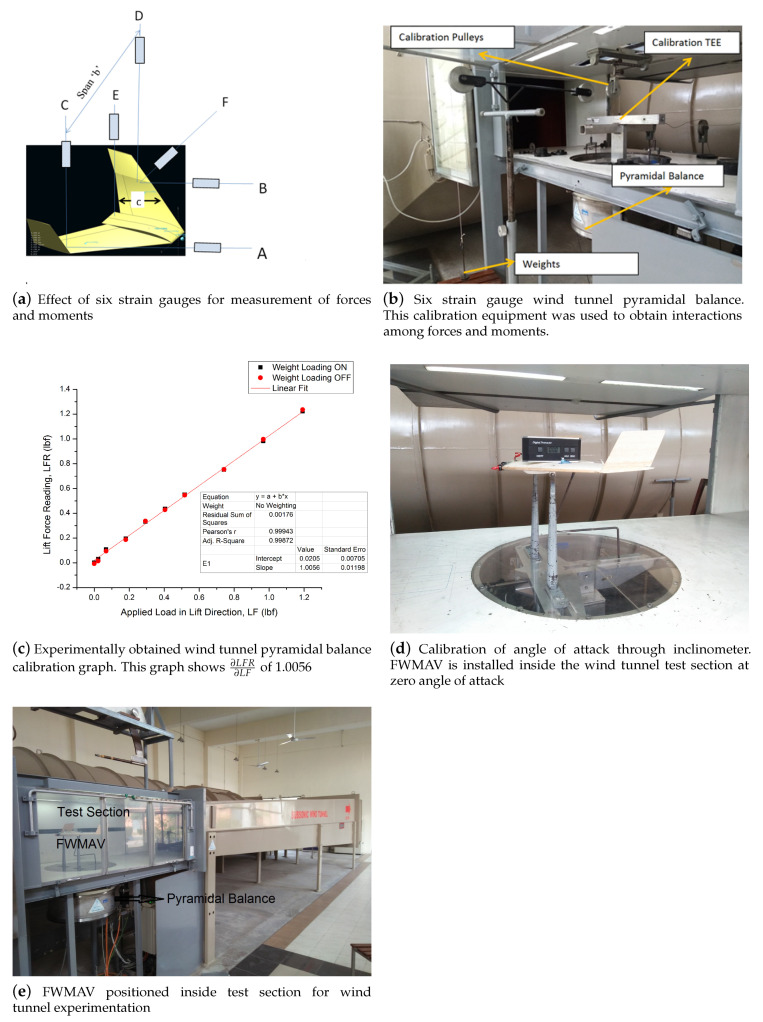
Wind Tunnel Calibrations.

Angle of attack is shown on the computer screen of a wind tunnel through a data acquisition system that is calibrated using an inclinometer placed on the top of a test model; refer to Figure 5d. When two data sets, one set from data acquisition system and the other from actual measurement from inclinometer are compared, the root mean square of the pairwise differences of the two data sets can serve as a measure of how far on average the error is from zero. This data set in shown in Table 5, where RMS value comes out to be 0.0144, which depicts that the data acquisition system executes a dependable angle of attack inside the test section. Figure 5d shows the angle of attack calibration process where inclinometer was placed over test model (FWMAV) at zero degree angle of attack. The angle of attack during wind tunnel experiments was varied from $-5^{\circ }$ to $+18^{\circ }$.

### 3.3. Flow Quality Inside the Test Section

Obtaining a spatially standardized steady stream of air inside the test section is a prerequisite for any wind tunnel experiment. Various parameters must be identified and quantified like flow angularity, variation of pressure along test section, noise levels, treatment of boundary layer near test section walls and behavior of vortices generated from vertical walls’ corners. However, level of unsteady velocity fluctuations about average velocity known as turbulence level is considered to be most critical. Turbulent fluid motion is an irregular condition of flow in which various quantities reflect random variation with time and space. There are inherent instabilities in the flow which must be quantified. It has been observed that if there is high level of turbulence for laminar experiments, then there will be unfavorable transitions and measurements of lift, drag, and velocity profiles may be incorrect. A hot wire anemometery system provided by DANTEC dynamics was used to determine turbulent intensity of a subsonic wind tunnel. Single wire probe MiniCTA 54T42 type 55P16 was used for velocity measurements inside the test section which was placed 200 mm downstream of test section entry. The sensor is a tungsten wire with gold plated ends. Data were recorded using 16-bit 4 velocity channel NI 9215 A/D board with sampling frequency of 200 kHz using streamware software. Probe was calibrated using manual calibrator with variable flow velocities from 0 m/s to 40 m/s. Turbulence intensity of 0.25% was recorded at speeds less than 30 m/s.

### 3.4. Pyramidal Balance Calibration

Calibration was carried out to proof load the balance and to ascertain component sensitivity. Balance interactions and calibration slopes for each component were determined. Balance interactions were calculated by putting weights on TEE in all three forces and three moments’ directions. Measurement of drag force required considerable effort as drag force was comprised of interference and tare drags. Model supports also contributed additional drag and affected air flow pattern around the model. Drag of supports is called “tare drag” while variation in air flow pattern due to presence of supports is called “interference drag” [37]. Evaluation of these types of drag is a complicated matter; therefore, these are evaluated together in a two-step process. In first step, drag is calculated in the inverted position of model using Equation (4)

(4) $$D_{1}=D_{inverted}+T_{upper}+I_{upper}.$$

Here, $D_{inverted}$ is inverted drag of test model, $T_{upper}$ is tare drag and $I_{upper}$ is the interference drag on upper surface of test model. In a second step, dummy supports are added to an already installed inverted test model and drag is measured again. In this case, drag is calculated as shown in Equation (5):

(5) $$D_{2}=D_{inverted}+T_{upper}+I_{upper}+T_{lower}+I_{lower}.$$

Difference between Equations (4) and (5) yields sum of tare and interference drag on lower surface, which is required to be known and needs to be subtracted from model drag reduction data. Six strain gauges are installed on the pyramidal balance as shown in Figure 5a. Forces are calculated using different strain gauges as indicated, while moments are calculated by multiplying force indicated by strain gauge with perpendicular distance (moment arm). Lift force is calculated as summation of inputs from C,D,E strain gauges, whereas pitching moment is calculated by multiplying input from *E* strain gauges with chord length *c*. Exact perpendicularity was ensured with the help of inclinometer placed on top of a nylon fish line. Pulleys were placed at about center line height of the test section as shown in Figure 5b. Loads were added progressively in forces and moment directions and all six readings were noted from a data acquisition system. By doing this, the effect of each force or moment on other components was measured. Six component calibration matrices were used to correct results for cross coupled measurement error between three forces and three moments. Design of pyramidal balance is such that interactions of one force on other force cannot be ruled out. These interactions were determined during calibration of balance and shown in matrix form as:

$$\begin{array}{c} \left[\begin{array}{cccccc} \frac{\partial LFR}{\partial LF} & \frac{\partial LFR}{\partial DF} & \frac{\partial LFR}{\partial SF} & \frac{\partial LFR}{\partial PM} & \frac{\partial LFR}{\partial YM} & \frac{\partial LFR}{\partial RM}\\ \frac{\partial DFR}{\partial LF} & \frac{\partial DFR}{\partial DF} & \frac{\partial DFR}{\partial SF} & \frac{\partial DFR}{\partial PM} & \frac{\partial DFR}{\partial YM} & \frac{\partial DFR}{\partial RM}\\ \frac{\partial SFR}{\partial LF} & \frac{\partial SFR}{\partial DF} & \frac{\partial SFR}{\partial SF} & \frac{\partial SFR}{\partial PM} & \frac{\partial SFR}{\partial YM} & \frac{\partial SFR}{\partial RM}\\ \frac{\partial PMR}{\partial LF} & \frac{\partial PMR}{\partial DF} & \frac{\partial PMR}{\partial SF} & \frac{\partial PMR}{\partial PM} & \frac{\partial PMR}{\partial YM} & \frac{\partial PMR}{\partial RM}\\ \frac{\partial YMR}{\partial LF} & \frac{\partial YMR}{\partial DF} & \frac{\partial YMR}{\partial SF} & \frac{\partial YMR}{\partial PM} & \frac{\partial YMR}{\partial YM} & \frac{\partial YMR}{\partial RM}\\ \frac{\partial RMR}{\partial LF} & \frac{\partial RMR}{\partial DF} & \frac{\partial RMR}{\partial SF} & \frac{\partial RMR}{\partial PM} & \frac{\partial RMR}{\partial YM} & \frac{\partial RMR}{\partial RM} \end{array}\right] \end{array}$$

These relations lead to a 6×6 matrix in which all the diagonal elements represent slopes of calibration curves for lift force, drag force, side force, pitching moment, yawing moment, and rolling moment. The diagonal numbers appeared to be larger in values as compared to off diagonal numbers. This is because interaction of one force on other forces and moments is relatively small. In an ideal case where there are no interactions, calibration matrix would be identity matrix of 6×6. Since wind tunnel used in this research does not have an ideal measuring apparatus, interactions therefore appeared, which generated a calibration matrix. Balance constants are obtained by an inversion of calibration matrix. For instance, ‘LFR’ stands for lift force reading from DAQ system, while ‘LF’ means applied load in lift force direction. A graph of lift force reading versus lift force has a slope which is represented as $\frac{\partial LFR}{\partial LF}$. This graph is shown in Figure 5c. Pearson’s correlation coefficient of the graph is 0.99943. Pearson’s correlation coefficient or Pearson’s r or bivariate correlation is a statistic tool that measures linear correlation between two variables like LFR (lift force reading) and LF (applied lift force) in this research. It is added that correlation reflects the strength and direction of a linear relationship, but not the slope of the relationship. 0.99943 on the scale of 1.000 reflected that lift force reading values increase with applied load in force direction. This scheme of calibration generated 36 interaction graphs, three forces and three moments with three forces and three moments, which will make a 6×6 matrix. The slope of every graph will represent one place in a calibration matrix. The calibration matrix is shown where all partial derivatives are placed at their designated places. These 36 graphs showed an interaction of one particular force with other forces and moments. These graphs are placed as Appendix A to this research paper for completeness. Every graph has two lines which represent weight loading ‘ON’ and weight loading ‘OFF’ conditions. Weight loading ‘ON’ condition is one in which weights are progressively added on a pulley mechanism and readings are taken from a wind tunnel data acquisition system while Weight loading ‘OFF’ conditions represent the scenario once weight are progressively removed from a pulley mechanism. The slope of every graph is then fed into a calibration matrix to take the place of partial derivatives in a calibration matrix. The inversion of calibration matrix yielded balance constants. These balance constants are then multiplied with forces and moments readings from data acquisition system to get final readings of forces and moments.

## 4. Results of Wind Tunnel Experiments

Wind Tunnel Tests of Fixed Wing Micro Aerial Vehicle of scale 1:1 were conducted at varying angles of attack from $-5^{\circ }$ to $+18^{\circ }$ for Reynolds numbers corresponding to free stream velocities of 20 m/s. Testing velocity of 20 m/s is chosen since lift equals weight condition was computationally predicted by XFLRv5 analysis at velocity of 18.5 m/s. Therefore, wind tunnel tests were conducted at a little higher velocity. The wind tunnel is well suited for static force testing on any aerodynamic body. Strain gauge pyramidal balance is used for measuring forces and moments on complete FWMAV as shown in Figure 5e.

### 4.1. Coefficient of Lift

**Figure 6:**
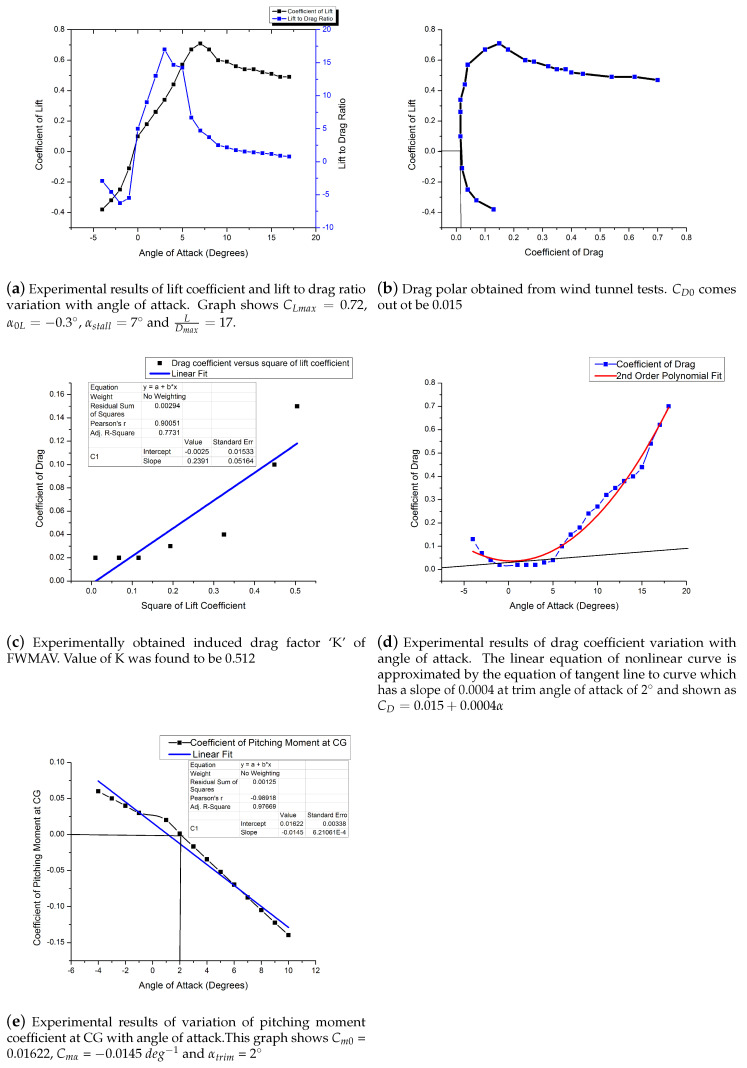
Experimentally obtained wind tunnel results.

Wind tunnel tests were conducted for full scale at Reynolds number of $3.25\times 10^{5}$ at velocity of 20 m/s. Coefficient of lift versus angle of attack was obtained through wind tunnel measurements and shown in Figure 6a. The $C_{L\alpha }$ was found to be 0.0552 per degree when values were taken till angle of attack of $8^{\circ }$. Theoretically, $C_{L\alpha }$ for FWMAV was calculated by formula proposed by H.B. Helmbold for low aspect ratio straight wings [38]. However, a more accurate approximation for a swept wing was suggested by Kuchemann [39], shown as Equation (6). Kuchemann suggested lift curve slope for an infinite swept wing be $a_{0}cos\Lambda$, where $a_{0}$ is the lift slope for airfoil section perpendicular to leading edge and Λ is the wing leading edge sweep. Once $a_{0}$ is replaced with $a_{0}cos\Lambda$, the Helmbold’s equation resulted in Kuchemann’s equation. Lift curve slope was calculated using Kuchemann’s formula; it came out to be 0.0523 per degree against a value of 0.0552 per degree from wind tunnel experiments. XFLRv5 was also used to calculate lift curve slope and it came out to be 0.0499 per degree. Error between theoretical and XFLRv5 results came out to be 4.58%, whereas error between theoretical and wind tunnel experiments came out to be 5.23%. Since FWMAV was designed with high leading edge sweep angle, perfect stalling characteristics were found missing in $C_{L}$ versus α curve [40]. Highly swept wings stall at very high angles of attack [41]. $C_{Lmax}$ of 0.72, $\alpha_{0L}$ of $-0.3^{\circ }$ and $\alpha_{stall}$ of $7^{\circ }$ are obtained graphically from Figure 6a:

(6) $$a=\frac{a_{0}cos\Lambda }{\sqrt{1+{\left(\frac{a_{0}cos\Lambda }{\pi AR}\right)}^{2}}+\frac{a_{0}cos\Lambda }{\pi AR}}.$$

### 4.2. Drag Polar

As a result of wind tunnel experiments, drag coefficient variation with lift coefficient is shown in Figure 6b. Values of wind tunnel $C_{D}$ represented true values of drag coefficient, since wind tunnel tests were conducted at full scale Reynolds number and no scaling effects were needed in experiments. $C_{D}$ behavior with α as obtained is shown in Figure 6d. Nonlinear behavior of $C_{D}$ curve with angle of attack is typical and is used to calculate induced drag coefficient factor *k*, once experimentally obtained ${\left(C_{L}\right)}^{2}$ values were plotted against $C_{D}$; refer to Figure 6c. Induce drag coefficient factor (K) of 0.2391 and Ostwald’s efficiency factor (e) of 0.512 were calculated. This value is away from the traditional value of 0.95 as found in literature. This is the peculiar problem of low aspect ratio wings which result in a nontraditional value of (e). $C_{D0}$ occurs at a very close value to the $C_{Dmin}$ point due to reflexed camber in E-387 airfoil. Increase in drag at higher values of angle of attack is due to flow separation phenomenon. With the knowledge of induce drag coefficient factor (K) and $C_{D0}$, drag polar equation is presented as $C_{D}=0.015+0.512{\left(C_{L}\right)}^{2}$. The linear equation of this nonlinear curve is approximated by the equation of tangent line to curve, which has a slope of 0.0004 at trim angle of attack of $2^{\circ }$. This linearization is valid for a small range of alpha on both sides of the intersection point. The linear equation of drag coefficient can be written as $C_{D}=0.015+0.0004\alpha$.

### 4.3. Lift to Drag Ratio

Experimentally obtained lift to drag ratio is plotted versus angle of attack in Figure 6a. $\frac{L}{D_{max}}$ of 17 was obtained at angle of attack of $3^{\circ }$. The behavior of L/D is typical of cambered wing where initially it increases and then decreases after reaching its maximum value ([42]).

### 4.4. Longitudinal Stability

Longitudinal static stability requirements dictated negative value of $C_{m\alpha }$ which represented change in pitching moment (about FWMAV center of gravity) with change in angle of attack. Wind tunnel results obtained for $C_{m}$ versus α are shown in Figure 6e, which has a negative slope of −0.0145 per degree, thereby confirming longitudinal stability in tested conditions. CG location was kept at 23% MAC (Mean Aerodynamic Chord) with zero elevon deflections. Since center of pyramidal balance and CG of FWMAV were not aligned, moment at center of balance was therefore shifted to CG and then converted into non-dimensional form to get coefficient of pitching moment at CG as shown in Equation (7). $C_{m0}$ was calculated at an angle of attack $0^{\circ }$ as 0.01622. The linear equation of pitching moment coefficient wind tunnel data can be stated as $C_{m}=C_{m0}+C_{m\alpha }\alpha =0.01622-0.0145\alpha$.

(7) $$M_{CG}=M+L\cdot X.$$

## 5. Elevon Control Derivatives

Conventional control surfaces of an aerial vehicle are considered to be elevator for pitch control, aileron for roll control and rudder for yaw control [21]. Airplane must have sufficient control power to maintain steady state flight and to safely maneuver from one steady state to another state. Proposed FWMAV incorporates elevon control surfaces for pitch and roll control, instead of elevators and ailerons. The directional stability of FWMAV is catered by winglets which do not have any moveable surface like rudder.

Since wind tunnel tests were not carried out for varying elevon deflections due to complexity of control deflection involved, therefore XFLRv5 potential flow solver was used for determination of control derivatives $C_{L\delta e}$, $C_{m\delta e}$, $C_{D\delta e}$, $C_{Y\delta e}$, $C_{l\delta e}$ and $C_{n\delta e}$. XFLRv5 gives three option for analysis purposes, which are LLT (Lifting Line Theory), panel methods and VLM(Vortex Lattice Method). LLT does not give accurate results for low aspect ratio wings with large amount of sweep and dihedral angles [43]. Since FWMAV has all these geometrical features, LLT option was therefore not selected in XFLRv5 for analysis of FWMAV with varying elevon deflections. The major benefit for the panel method is inclusion of fuselage effect in calculations of forces and moments. FWMAV is a flying wing configuration; therefore, no additional benefit is obtained by using the panel method. Additionally, the panel method is used where coefficient of pressure distribution is to be found on top or bottom surfaces. VLM is applicable to any wing geometry including sweep, dihedral and low aspect ratios [44]. In VLM, lift force is calculated using the Kutte–Joukowski formula written as L=ρ·V·Γ.

**Figure 7:**
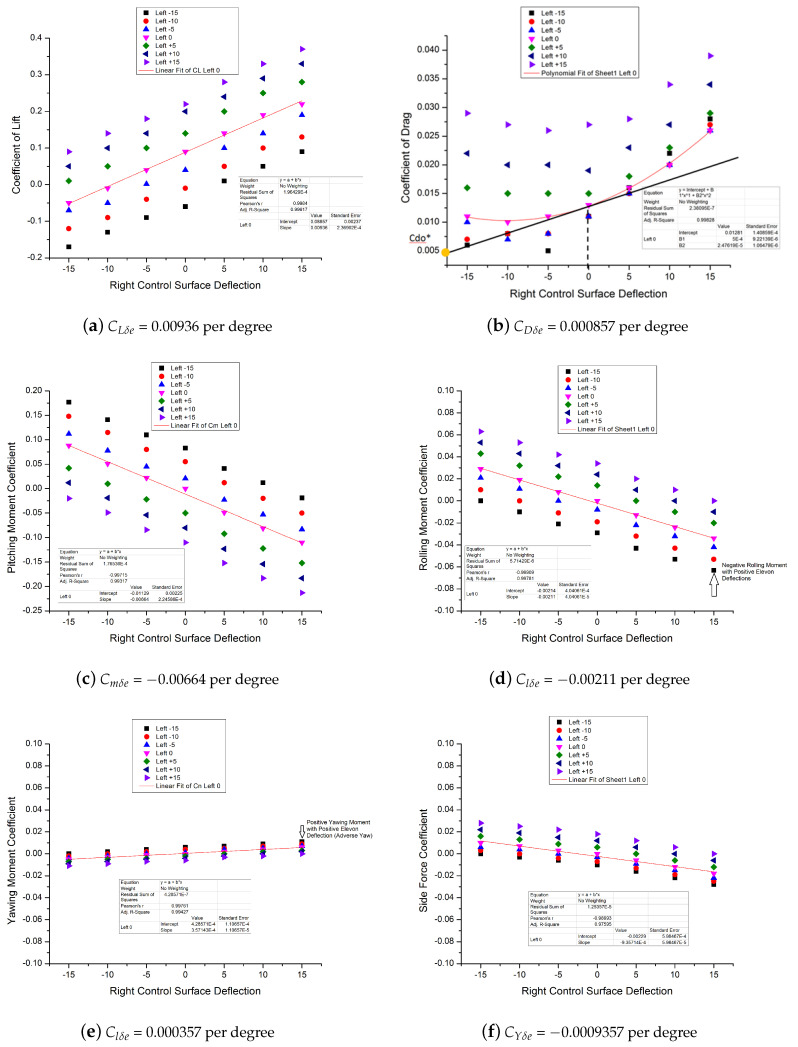
Computationally calculated elevon control derivatives. Derivatives are calculated from linear curve fit passing from zero control deflection.

For determination of control derivatives, a total of 49 configurations were computationally modeled in XFLRv5 with varying elevon control deflections from $-15^{\circ }$ to $+15^{\circ }$ with a difference of $5^{\circ }$. These configurations were analyzed using type 2 (lift equals weight conditions) and type 7 (stability analysis). For details of XFLRv5 analysis conditions, the reader is requested to review [45]. The values of force and moment coefficients were noted against trim angle of attack of $2^{\circ }$. Trim angle of attack was noted from pitching moment coefficient graph, where $C_{m}$ values intersect the angle of attack axis; refer Figure 6e. The data are handled in a way to dig out slopes of curves which, in this case, are the control derivatives. Graphs are plotted for forces and moments coefficients against elevon control deflections. It can be seen from Figure 7a–c that curves have the same trend with elevon deflections, as with angle of attack. Lift coefficient has a linear line curve with positive slope and pitching moment coefficient has linear curve with negative slope. Shifting of curves with changing elevon deflections can also be noticed. From the results of Figure 7a,c, $C_{L\delta e}$ was found to be $0.00936\deg^{-1}$, whereas $C_{m\delta e}$ came out to be $-0.00664\deg^{-1}$.

From Figure 7b, drag coefficient change with elevon deflection has a typical nonlinear behavior, like drag variation with angle of attack. The nonlinearity in Figure 7b is dealt via linearization around trim point using small disturbance theory ([21]). The linear equation of this nonlinear curve is approximated by the equation of tangent line to curve which has slope of 0.000857 for elevon deflection $\delta_{e}$ of $0^{\circ }$ at trim angle of attack of $2^{\circ }$. The pictorial representation is shown in Figure 7b and the equation can be modeled as $C_{D}=0.005+0.000857\cdot \delta_{e}$.

Rolling moment coefficient change with elevon deflection (Figure 7d), yawing moment change with elevon deflection (Figure 7e) and side force coefficient change with elevon deflection (Equation (16)) are calculated as $C_{l\delta e}=-0.00211\deg^{-1}$, $C_{n\delta e}=0.000357\deg^{-1}$, and $C_{Y\delta e}=-0.000935\deg^{-1}$, respectively. As can be seen from numerical values, FWMAV yawing moment coefficient and side force coefficient change with elevon deflections are one order of magnitude less as compared to rolling moment coefficient. Rolling moment coefficient is negative with positive deflection of elevon, which is consistent with the convention of control derivatives. Sign conventions regarding control derivatives as described in airplane dynamics text books are adopted in this research work [32].

From Figure 7d,e, it is observed that minor adverse yaw is noticed with change in positive elevon deflections. Positive yawing moment is created with negative rolling moment. However, the magnitude of adverse yaw is not significant which was evident during flight trials as MAV successfully performed coordinated turns quite well without any handling problems. From Figure 7e, it is observed that, since no dedicated vertical control surface was designed; therefore, $C_{n\delta e}$ was found to be one order of magnitude smaller as compared to $C_{l\delta e}$ and $C_{m\delta e}$.

After obtaining control derivatives from potential flow solver (XFLRv5) and aerodynamic coefficients from wind tunnel tests, a 6-DoF model will be implemented in the next section.

## 6. Implementation of a 6-DoF Model

Six Degree of Freedom equations are essentially the application of Newton’s second law of motion in translational and rotational motions using an inertial frame of reference. Newton’s second law states that applied forces cause the rate of change of linear momentum ($F=\frac{d(mv)}{dt}$) and applied moments cause the rate of change of angular momentum ($Moments=\frac{dH}{dt}$). Angular momentum is defined as moment of linear momentum. It is appreciated that Newton’s law is applicable to inertial frame of reference only. For application of this law on earth for a flying wing micro aerial vehicle, the earth is considered to be flat and non-rotating, which can make it the inertial frame. Rigid body is free to change position in translational axis combined with changes in orientation through rotation about three perpendicular axes. These three rotations are often termed as yaw, pitch and roll. 6-DoF equations are written in a body fixed axis system, and it is assumed that gravitational and thrust vectors are acting at the center of gravity, thereby producing no moment. ϵ is the angle of thrust vector with body longitudinal axis, whereas θ is the pitch angle with respect to horizontal:

(8) $$\dot{u}=rv-qw-C_{x}\cdot \frac{q_{\infty }S}{m}-gsin\theta +\frac{Tcos\epsilon }{m}$$

(9) $$\dot{v}=pw-ru-C_{y}\cdot \frac{q_{\infty }S}{m}+gcos\theta sin\phi$$

(10) $$\dot{w}=pv-qu-C_{z}\cdot \frac{q_{\infty }S}{m}+gcos\theta cos\phi -\frac{Tsin\epsilon }{m}$$

(11) $$L=I_{xx}\dot{p}-I_{xz}\dot{r}+qr\left(I_{zz}-I_{yy}\right)-I_{xz}pq$$

(12) $$M=I_{yy}\dot{q}+rp\left(I_{xx}-I_{zz}\right)+I_{xz}\left(p^{2}-r^{2}\right)$$

(13) $$N=I_{zz}\dot{r}-I_{xz}\dot{p}+pq\left(I_{yy}-I_{xx}\right)+I_{xz}qr$$

The fundamental equations of motion are often stated as Equations (8)–(13). $C_{x}$, $C_{Y}$ and $C_{z}$ are coefficients of X-force, Y-force and Z-force defined in a body axis system. These coefficients can be written as $C_{x}=C_{D}cos\alpha -C_{L}sin\alpha$ and $C_{z}=C_{D}sin\alpha +C_{L}cos\alpha$. Upon implementation of small angle approximation, $C_{x}=C_{D}-C_{L}\alpha$ and $C_{z}=C_{D}\alpha +C_{L}$. Since $C_{L\alpha }$ and $C_{D\alpha }$ have very small magnitude, $C_{x}$ can therefore be estimated as $C_{D}$ and $C_{z}$ as $C_{L}$:

(14) $$C_{L}=C_{L0}+C_{L\alpha }\cdot \alpha +C_{Lu}\cdot \left(u/U\right)+C_{L\dot{\alpha }}\cdot \dot{\alpha }+C_{Lq}\cdot \left(qc/2U\right)+C_{L\delta e}\cdot \delta e$$

(15) $$C_{D}=C_{D0}+C_{D\alpha }\cdot \alpha +C_{Du}\cdot \left(u/U\right)+C_{D\delta e}\cdot \delta e$$

(16) $$C_{Y}=C_{Y0}+C_{Y\beta }\cdot \beta +C_{Y\delta e}\cdot \delta e$$

(17) $$C_{l}=C_{l0}+C_{l\beta }\cdot \beta +C_{lp}\cdot \left(pb/2U\right)+C_{lr}\cdot \left(rb/2U\right)+C_{l\delta e}\cdot \delta e$$

(18) $$C_{m}=C_{m0}+C_{m\alpha }\cdot \alpha +C_{m\dot{\alpha }}\cdot \dot{\alpha }+C_{mq}\cdot \left(qc/2U\right)+C_{m\delta e}\cdot \delta e$$

(19) $$C_{n}=C_{n0}+C_{n\beta }\beta +C_{np}\cdot \left(pb/2U\right)+C_{nr}\cdot \left(rb/2U\right)+C_{n\delta e}\delta e$$

The expressions for aerodynamic coefficients are mentioned in Equations (14)–(16) (refer [22]). It is appreciated that, for steady (un-accelerated), level (ϕ=0) and straight (θ=0) flight conditions, the LHS of force Equations (8) and (10) become zero. Since body does not have any rotational vector, Coriolis acceleration terms on RHS will also therefore be zero. In this scenario with zero thrust vector deflection (ϵ=0) with respect to body *x*-axis, Equations (8) and (10) become T=D and L=W respectively.

There exists an x–z plane of symmetry in FWMAV; therefore, products of inertia ($I_{yz}$ and $I_{xy}$) are zero. With this assumption, moment equations can be stated as shown in Equations (11)–(13). Here, L, M, and N represent rolling, pitching and yawing moment in a body fixed axis system. The rolling moment is defined as $L=C_{l}\cdot q\cdot S\cdot c$, pitching moment is defined as $M=C_{m}\cdot q\cdot S\cdot b$ and yawing moment is defined as $N=C_{n}\cdot q\cdot S\cdot b$. The moment coefficients are defined in Equations (17)–(19) (refer to [22]). $I_{xx}$, $I_{yy}$, and $I_{zz}$ are mass moment of inertia in body *x*-axis, body *y*-axis, and body *z*-axis, respectively. $I_{xz}$ is the product of mass moment of inertia. The estimation of mass moment of inertia is done in XFLRv5 software where care was taken in placement of exact components weight in correct places.

Aerodynamic static coefficients are evaluated using wind tunnel tests data and are extracted using Figure 6a–e. Control derivatives are evaluated using potential flow software results which were plotted in Figure 7a–f. Other derivatives like rate derivatives ($C_{Lq}$, $C_{lp}$, $C_{lr}$, $C_{np}$ and $C_{nr}$) are evaluated using XFLRv5 software for zero elevon deflection configuration. It is highlighted that u and $\dot{\alpha }$ derivatives (although mentioned in equations for completeness) are taken as zero. Since lift and drag do not vary with ‘u’ velocity in subsonic regime, their derivatives $C_{Lu}$ and $C_{Du}$ can be neglected for slow moving vehicles like FWMAV ([22]. Furthermore, there is no horizontal tail designed in FWMAV, so there will not be any lag in wing tip vortices reaching horizontal tail and therefore there will not be any lift or moment generated due to $\dot{\alpha }$, hence $\dot{\alpha }$ derivatives can also be neglected:

(20) $$\dot{u}=rv-qw-\left(0.015+0.000857\alpha +0.00055\delta e\right)\cdot \left(\frac{q_{\infty }S}{m}\right)-gsin\theta +\frac{Tcos\epsilon }{m}$$

(21) $$\dot{v}=pw-ru-\left(-0.00473\beta -0.0009357\delta e\right)\cdot \left(\frac{q_{\infty }S}{m}\right)+gcos\theta sin\phi$$

(22) $$\dot{w}=pv-qu-\left(0.1+0.0523\alpha +0.00035q+0.00936\delta e\right)\cdot \left(\frac{q_{\infty }S}{m}\right)+gcos\theta cos\phi -\frac{Tsin\epsilon }{m}$$

(23) $$-0.0147\beta -0.000057p+0.000016r-0.02179\delta e=0.000504\dot{p}+0.000023\dot{r}+0.000501qr+0.000236pq$$

(24) $$0.3413-0.054\alpha -0.000714q-0.026\delta e=0.0005845\dot{q}-0.0005817rp-0.0000236\left(p^{2}-r^{2}\right)$$

(25) $$0.002\beta +0.000013p-0.000024r+0.000003\delta e=0.001086\dot{r}+0.000023\dot{p}+0.00008pq-0.000023qr$$

In this scenario, 6-DoF equations of motion for flying wing micro aerial vehicles are stated in Equations (20)–(25). It is observed from the values of $C_{lp}$, $C_{lr}$,$C_{np}$,$C_{nr}$ in Equations (23) and (25) that these are three orders of magnitude smaller as compared to other aerodynamic derivatives. This depicts a small amount of roll that causes yaw and yaw causes roll phenomenon in fixed wing micro aerial vehicles. It is highlighted that propeller effects on low aspect ratio wings have been studied earlier through wind tunnel experimentation by various researchers [46,47,48,49]. However, for correct six degree of freedom modeling applications, it is recommended that dependency of aerodynamic coefficients on advance ratios rather than propeller rotation may also be studied.

## 7. Flight Tests

**Figure 8:**
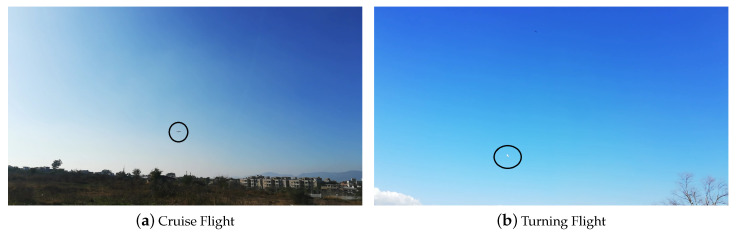
Flight tests of FWMAV. This figure shows successful flight during cruise and turning mission segments.

After fabrication, flight tests were conducted to ensure correct airfoil selection and to identify any design flaws for stable flight. Initially, FWMAV flew unsuccessful flights where stability was a major concern. The most probable cause was attributed to control surfaces, loose connecting rods and lower hand launch speed. Excessive play in connecting rods was removed by installing rods of larger diameter. The hand launch speed was increased. Complete details of probable cause for the failure of flight tests and remedial actions taken are mentioned against each flight in Appendix B. The majority of problems concerning unstable flight were related to excessive left rolling tendency and abrupt pitching motion. It was concluded that left rolling tendency was caused by clockwise rotation of propeller due to torque effect and abrupt pitching motion was attributed to gyroscopic precession [50]. After necessary modification of control rods, FWMAV successfully fulfilled its designed mission (Figure 1) and flew for 25 min until complete discharge of battery. Successful flight is shown in Figure 8.

## 8. Conclusions

In this research, an airfoil selection procedure for flying wing micro aerial vehicles has been proposed. Airfoil was selected based on highest value of proposed parameter which was calculated by non-dimensionalizing aerodynamic and stability coefficients. FWMAV with Eppler-387 airfoil was fabricated using styrofoam and static aerodynamic coefficients were calculated using wind tunnel experimentation. Exhaustive wind tunnel pyramidal balance calibration data were presented before data extraction. Control derivatives were determined by analyzing various elevon control deflections in XFLRv5. Using aerodynamic derivatives, a six Degree of Freedom model has been implemented. Flight tests presented major problems of left rolling tendency and abrupt yawing motions while pitching. Research concluded that these problems are related to torque effect of clockwise rotating propeller and gyroscopic precession.

This work was based on 10 reflexed airfoils, whereas procedure can be extended to include more airfoils for better data analysis. Inclusion of airfoils’ natural frequencies both in stationary and rotating conditions may be added before final selection of airfoil [51]. 

## Figures and Tables

**Table 1:** Numerically calculated aerodynamic and stability parameters of ten reflexed airfoils by XFLRv5 software.

Airfoil	$\frac{C_{l}}{C_{\mathrm{dmax}}}$	$\frac{{C_{l}}^{3/2}}{C_{\mathrm{dmax}}}$	$\frac{{C_{l}}^{1/2}}{C_{\mathrm{dmax}}}$	$C_{l0}$	$C_{\mathrm{lmax}}$	$\alpha_{\mathrm{stall}}$	$C_{l\alpha }$	$C_{d0}$	$C_{m\alpha }$	$C_{m0}$
E184	44.04	35.88	54.44	0.17	0.88	9	0.0856	0.014	0.0058	−0.000124
E186	45.76	41.30	50.71	0.14	0.98	10.5	0.0960	0.021	0.0034	**−0.000042**
E387	**60.90**	**65.98**	56.28	**0.42**	**1.35**	11.75	**0.1028**	0.015	0.0043	−0.000141
FX69H083	53.07	49.18	**57.27**	0.28	1.05	9.5	0.0439	0.015	0.0022	−0.000451
M5	46.73	42.04	52.27	0.21	1.03	10	0.0928	0.013	0.0039	−0.000471
M6	45.56	49.78	48.04	0.30	1.23	**13.5**	0.0906	0.017	**0.0058**	−0.000251
S5010	45.07	44.29	48.13	0.20	1.25	12	0.0951	0.014	0.0025	−0.000133
S5020	46.08	45.27	49.26	0.21	1.21	11	0.1005	0.014	0.0021	−0.000151
MH60	45	43	42.28	0.23	1.20	10.5	0.099	0.015	0.0031	−0.000711
HS-522	44.5	42.51	42.64	0.23	1.19	11	0.091	**0.012**	0.0025	−0.000859

**Table 2:** τ, η, and Σ values for ten reflexed airfoils. The table shows Eppler-387 has best overall performance in terms of τ, η, and Σ.

Airfoil	τ	η	Σ
E184	10.44	44.92	27.68
E186	10.13	45.96	28.045
E387	14.42	61.08	37.75
FX69H083	12.11	53.19	32.65
M5	9.82	47.07	28.445
M6	11.82	48.24	30.03
S5010	9.37	45.98	27.675
S5020	9.33	47.03	28.18
MH60	9.02	43.11	26.065
HS-522	8.41	42.96	25.685

**Table 3:** Geometric features of flying wing micro aerial vehicle.

Nomenclature	Specifications
Airfoil	Epper-387 (reflexed)
Planform	Rectangular Swept
Leading Edge Sweep	$40^{\circ }$
Trailing Edge Sweep	$20.43^{\circ }$
Propeller Diameter	126.31 mm
Span	499.87 mm
Taper Ratio	0.5
Winglet Taper Ratio	0.6

**Table 4:** Wind tunnel velocity calibration. Velocity inside the test section was calibrated with pitot-static tube and inclined air meter.

Air Meter (m/s)	Total Pressure (in)	Static Pressure (in)	ΔH (in)	ΔH (m)	Velocity (m/s)	% Error
22.35	16.8	18.3	1.5	0.0381	22.242	0.485
17.88	17.2	18.2	1.0	0.0254	18.053	0.958
14.01	17.5	18.1	0.6	0.0152	14.048	0.27
9.82	17.7	18.0	0.3	0.0076	9.933	1.137
5.64	17.8	17.9	0.1	0.00254	5.742	1.776

**Table 5:** Angle of attack calibration. Angle of attack was calibrated against inclinometer and data acquisition system of wind tunnel.

Inclinometer Reading	DAQ Reading	Difference	Square of Difference
−5	−4.99	0.01	1 × 10${}^{-4}$
−3	−3.01	0.01	1 × 10${}^{-4}$
−1	−1.01	0.01	1 × 10${}^{-4}$
1	1.02	0.02	4 × 10${}^{-4}$
3	2.99	0.01	1 × 10${}^{-4}$
5	5.01	0.01	1 × 10${}^{-4}$
7	6.99	0.01	1 × 10${}^{-4}$
9	9.01	0.01	1 × 10${}^{-4}$
11	11.01	0.01	1 × 10${}^{-4}$
13	12.98	0.02	4 × 10${}^{-4}$
15	14.99	0.01	1 × 10${}^{-4}$
17	16.98	0.02	4 × 10${}^{-4}$

**Figure A1:**
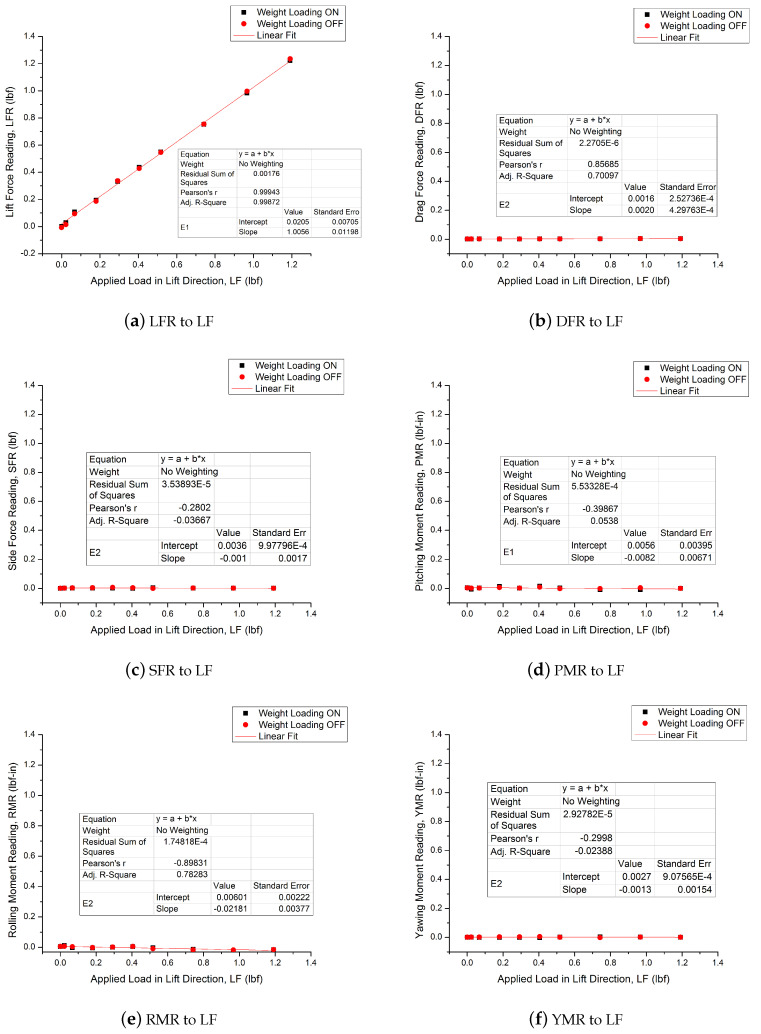
Experimentally obtained wind tunnel pyramidal balance interactions upon applied load in lift force direction.

**Figure A2:**
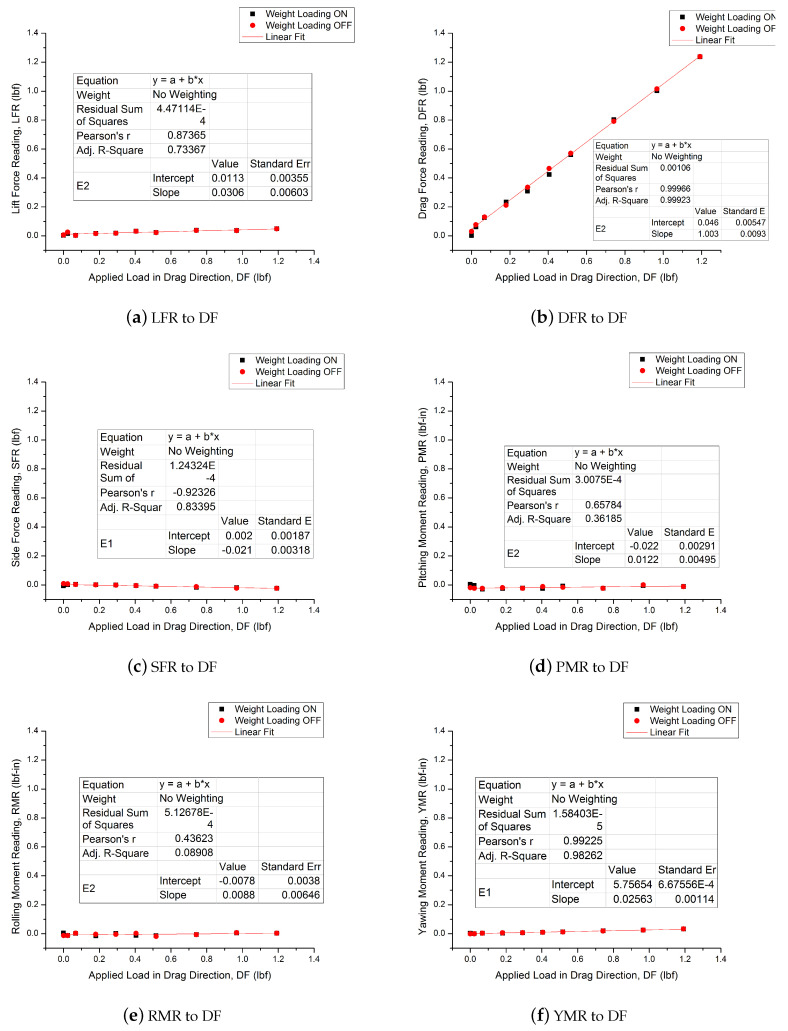
Experimentally obtained wind tunnel pyramidal balance interactions upon applied load in drag force direction.

**Figure A3:**
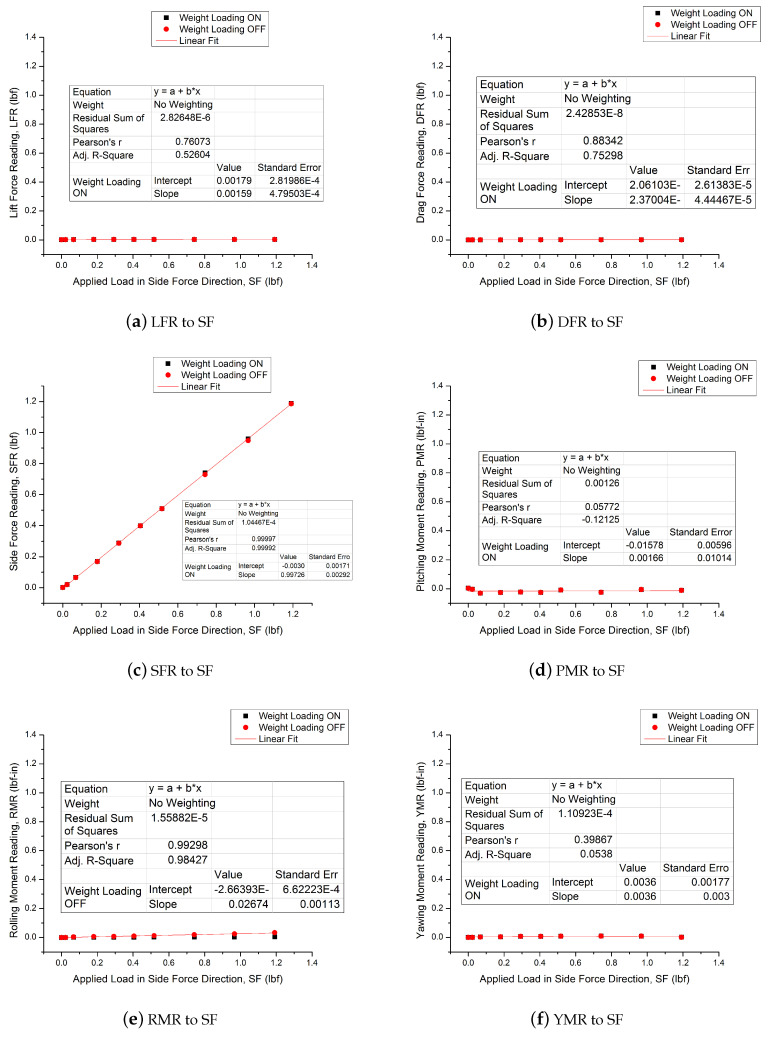
Experimentally obtained wind tunnel pyramidal balance interactions upon applied load in side force direction.

**Figure A4:**
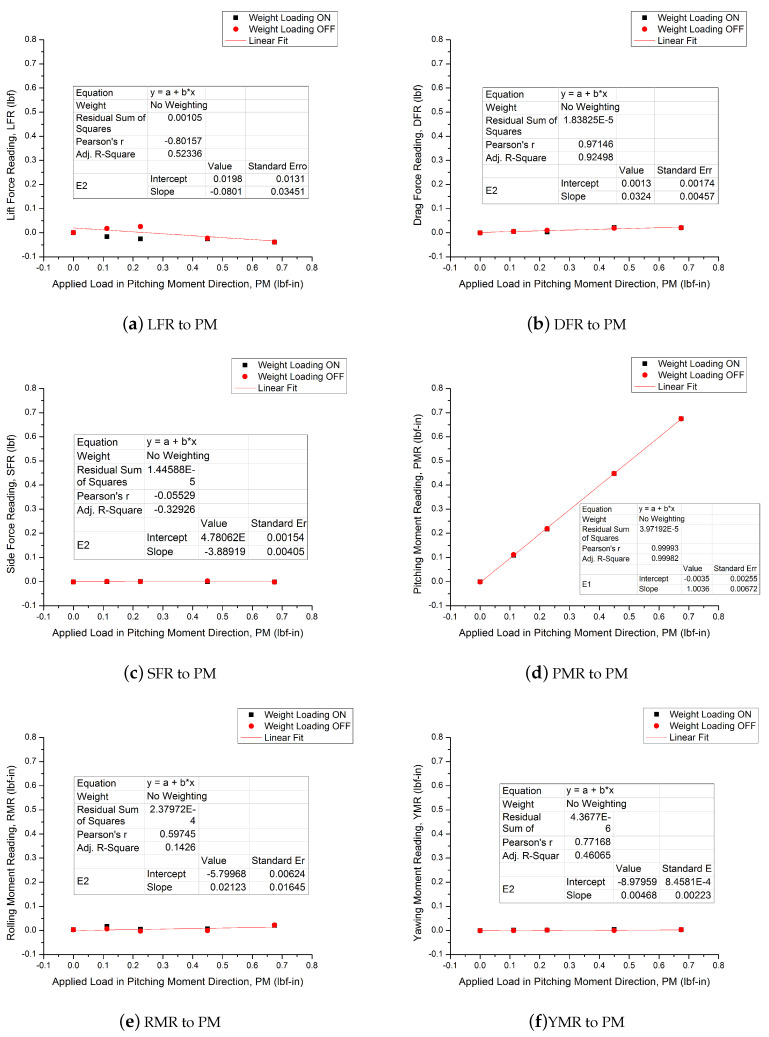
Experimentally obtained wind tunnel pyramidal balance interactions upon applied load in pitching moment direction.

**Figure A5:**
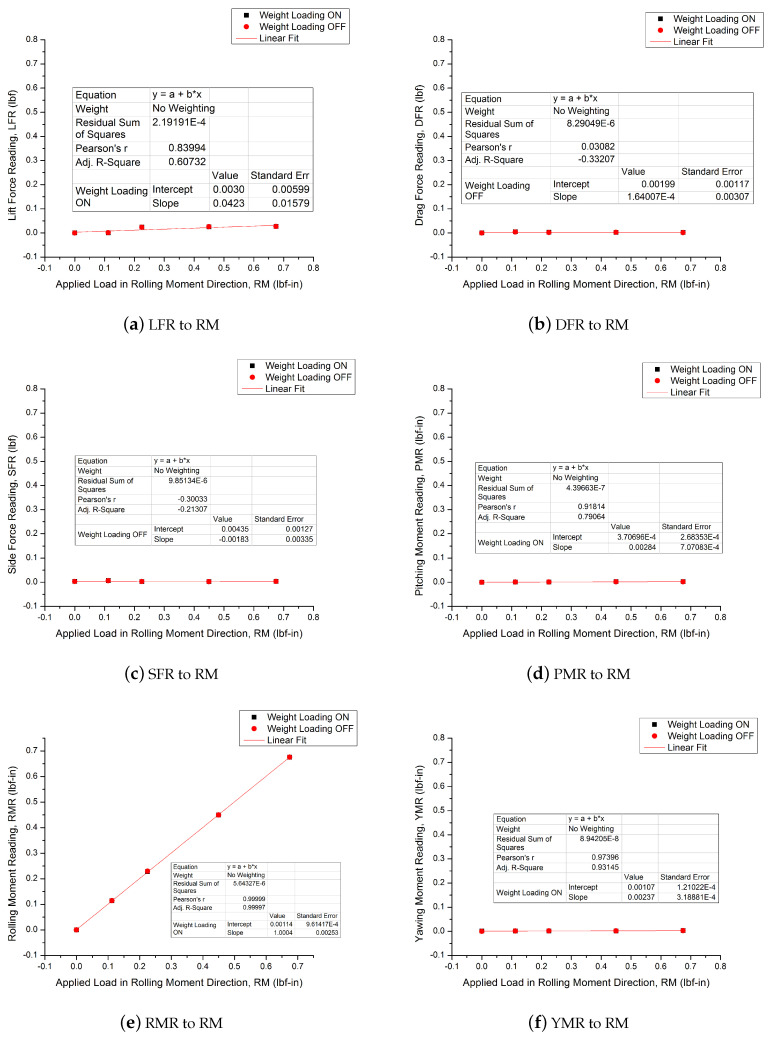
Experimentally obtained wind tunnel pyramidal balance interactions upon applied load in rolling moment direction.

**Figure A6:**
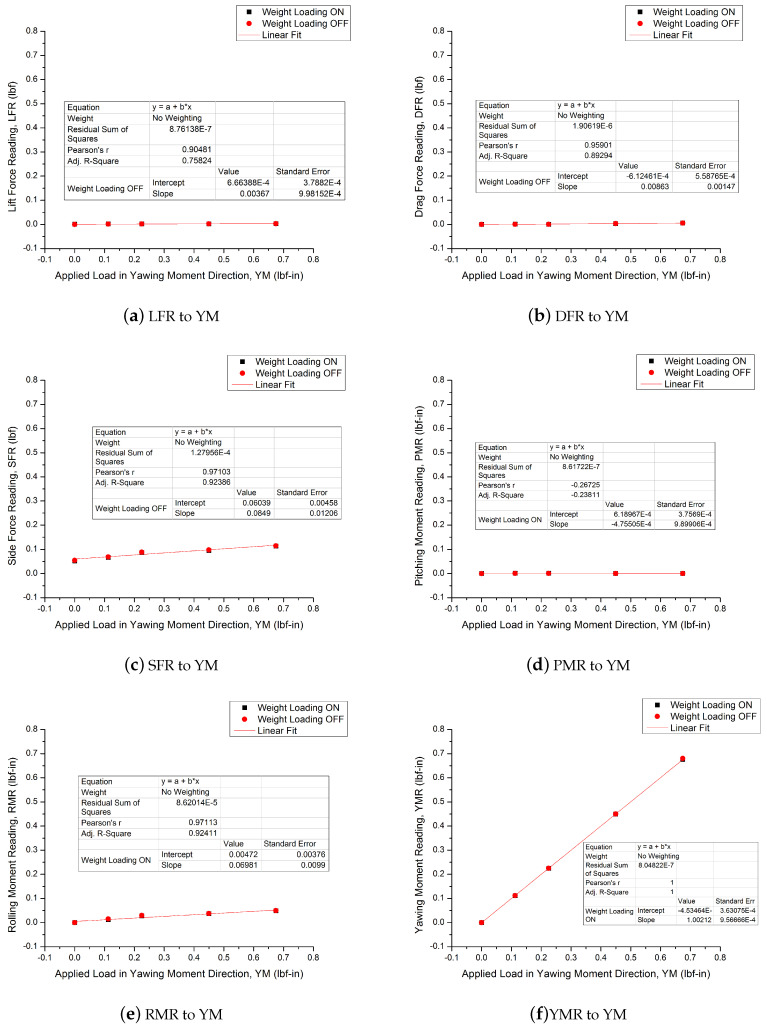
Experimentally obtained wind tunnel pyramidal balance interactions upon applied load in yawing moment direction.

## Nomenclature

ARPA	Advanced Research Projects Agency
AR	Aspect ratio of wing
CFD	computational fluid dynamics
$D_{1}$	Summation of upper surface, tare and interference drag
$D_{2}$	Summation of $D_{1}$ and $T_{lower}$ and $I_{lower}$
$D_{inverted}$	Drag while test model is in inverted position
FWMAV	Flying wing micro aerial vehicle
*H*	Height of fluid inside manometer
IAS	indicated air speed
$I_{upper}$	Interference drag on upper surface of test model
$I_{lower}$	Interference drag on lower surface of test model
LLT	lifting line theory
OEM	Original Equipment Manufacturer
RAND	Research ANd Development
RSTA	Reconnaissance Surveillance and Target Acquisition
$T_{upper}$	Tare drag on upper surface of test model
$T_{lower}$	Tare drag on lower surface of test model
6DOF	six degrees of freedom
VLM	vortex lattice method
*X*	Distance from balance center to CG of FWMAV
*u*	component of velocity in *x*-body axis
*v*	component of velocity in *y*-body axis
*w*	component of velocity in *z*-body axis
*p*	roll rate
*q*	pitch rate
*r*	yaw rate
*L*	lift
*D*	drag
*Y*	side force
*L*	rolling moment
*M*	pitching moment
*N*	yawing moment
*U*	velocity vector
*q*	dynamic pressure
AR	wing aspect ratio
*b*	wing span
*S*	wing surface reference
$I_{xx}$	mass moment of inertia along *x*-body axis
$I_{yy}$	mass moment of inertia along *y*-body axis
$I_{zz}$	mass moment of inertia along *z*-body axis
$I_{xz}$	product of moment of inertia
$\bar{c}$	mean aerodynamic chord
ρ	density
α	angle of attack
β	side slip angle
η	airfoil performance parameter
Γ	circulation around wing
Λ	wing leading edge sweep angle (degrees)
τ	airfoil efficiency parameter
LFR	lift force reading
DFR	drag force reading
SFR	side force reading
PMR	pitching moment reading
YMR	yawing moment reading
RMR	rolling moment reading
$C_{L}$	lift coefficient
$C_{D}$	drag coefficient
$C_{Y}$	side force coefficient
$C_{l}$	rolling moment coefficient
$C_{m}$	pitching moment coefficient
$C_{n}$	yawing moment coefficient
$C_{L0}$	airplane lift coefficient for zero angle of attack
$C_{D0}$	airplane zero lift drag coefficient
$C_{m0}$	airplane pitching moment coefficient at zero angle of attack
$a_{0}$	Theoretical airfoil section lift curve slope
$C_{l\alpha }$	airfoil section lift coefficient variation with angle of attack
$C_{L\alpha }$	airplane lift coefficient variation with angle of attack
$C_{D\alpha }$	airplane drag coefficient variation with angle of attack
$C_{Y\beta }$	airplane side force coefficient variation with side slip angle
$C_{l\beta }$	airplane rolling moment coefficient variation with side slip angle
$C_{m\alpha }$	airplane pitching moment coefficient variation with angle of attack
$C_{n\beta }$	airplane yawing moment coefficient variation with side slip angle
$C_{Lq}$	airplane lift coefficient variation with dimensionless pitch rate
$C_{mq}$	airplane pitching moment coefficient variation with pitch rate
$C_{L\delta e}$	airplane lift coefficient variation with elevon deflection
$C_{D\delta e}$	airplane drag coefficient variation with elevon deflection
$C_{Y\delta e}$	airplane side force coefficient variation with elevon deflection
$C_{l\delta e}$	airplane rolling moment coefficient variation with elevon deflection
$C_{m\delta e}$	airplane pitching moment coefficient variation with elevon deflection
$C_{n\delta e}$	airplane yawing moment coefficient variation with elevon deflection
$C_{L\dot{\alpha }}$	airplane lift coefficient variation with rate of change of angle of attack
$C_{lp}$	airplane rolling moment coefficient variation with roll rate
$C_{lr}$	airplane rolling moment coefficient variation with yaw rate
$C_{np}$	airplane yawing moment coefficient variation with roll rate
$C_{nr}$	airplane yawing moment coefficient variation with roll rate

## Appendix B. Results of Flight Tests Conducted

**Date**	**Time of Flight (min)**	**Flight Status**	**Causes of Failure (If Any)**	**Remedial Action for the Next Flight**
5 Jan 2020	0.08	Flight failed	Low launch velocity and less pitch angle	Increase launch velocity and pitch
5 Jan 2020	0.08	Flight failed	Initial pitch angle was not sufficient	Increase takeoff pitch angle
5 Jan 2020	0.16	Flight failed	Throttle could not provide stabilized thrust at take off	Increase throttle
6 Jan 2020	0.58	Flight failed	Throttle could not provide stabilized thrust at take off	Full throttle takeoff
9 Jan 2020	1.2	Flight failed	Not enough thrust by propeller	Change battery
10 Jan 2020	2.65	Flight failed	Pitched down after takeoff	Trim elevons
11 Jan 2020	2.83	Flight failed	Pitched down after takeoff	Trim elevons
11 Jan 2020	2.95	Flight failed	Large oscillations in longitudinal mode after takeoff	Tighten control rods
12 Jan 2020	6.5	Level flight successful	Left rolling tendency	Elevon adjustment
13 Jan 2020	9.87	Level flight successful	Left rolling tendency	Tightening of control rods
13 Jan 2020	9.7	Level flight successful	Left rolling tendency	Elevon adjustment
14 Jan 2020	15	Flight Successful with minor adjustment	Left rolling tendency	Elevon adjustment
14 Jan 2020	15	Flight Successful with minor adjustment	Left rolling tendency	Replace control rods
14 Jan 2020	19	Flight Successful with minor adjustment	Left rolling tendency	Tightening of control rods
16 Jan 2020	21.5	Flight successful	Nil	Nil
16 Jan 2020	21	Flight successful	Nil	Nil
18 Jan 2020	24	Complete Mission successful	Nil	Nil
20 Jan 2020	22	Complete Mission successful	Nil	Nil

## Appendix C. Numerically Calculated Results of Experiments Conducted for S-5010 Airfoil at Reynolds Number of 200,000

**Exp No**	**Qty**	$-5^{\circ }$	$-4^{\circ }$	$-3^{\circ }$	$-2^{\circ }$	$-1^{\circ }$	$0^{\circ }$	$1^{\circ }$	$2^{\circ }$	$3^{\circ }$	$4^{\circ }$	$5^{\circ }$	$6^{\circ }$	$7^{\circ }$	$8^{\circ }$	$9^{\circ }$	$10^{\circ }$	$11^{\circ }$	$12^{\circ }$	$13^{\circ }$	$14^{\circ }$	$15^{\circ }$	$16^{\circ }$	$17^{\circ }$	$18^{\circ }$
1	**$\frac{C_{l}}{C_{d}}$**	−1.8	−1.3	0	2.13	7.9	13.6	19.13	24.3	29.44	34.4	39	42.8	**45.07**	42	38.1	33.4	28.8	23.2	17	12.1	9.83	9.7	9.5	9.4
2	**$\frac{{C_{l}}^{3/2}}{C_{d}}$**	0.3	0.2	0	0.3	2.6	6.1	10.4	15.2	20.6	32.4	38.1	42.4	**44.29**	43.5	40.9	36.8	32.2	25.8	18.6	12.8	10.1	10	9.7	9.6
3	**$\frac{{C_{l}}^{1/2}}{C_{d}}$**	10.8	9.4	0	12.4	23.5	30.3	35.1	38.8	42	44.7	46.9	**48.12**	47.5	44.9	40.6	35.6	30.4	25.7	20.9	15.6	11.4	9.5	9.4	9.2
4	**$C_{l0}$**	−0.03	−0.02	0	0.03	0.11	**0.20**	0.29	0.39	0.49	0.69	0.79	0.87	0.98	1.07	1.14	1.21	1.25	1.23	1.18	1.12	1.07	1.06	1.04	1.02
5	**$C_{lmax}$**	−0.03	−0.02	0.00	0.03	0.11	0.20	0.29	0.39	0.49	0.69	0.79	0.87	0.98	1.07	1.14	1.21	**1.25**	1.23	1.18	1.12	1.07	1.06	1.04	1.01
6	**$\alpha_{stall}$**	−5	−4	−3	−2	−1	0	1	2	3	4	5	6	7	8	9	10	11	**12**	13	14	15	16	17	18
7	**$C_{l\alpha }$**	0.1	0.1	0.1	0.1	0.1	0.1	0.1	0.1	0.1	0.1	0.1	0.1	0.1	0.1	0.1	0.1	0.1	0.1	0.1	0.1	0.1	0.1	0.1	0.1
8	**$C_{d0}$**	0.016	0.015	**0.014**	0.014	0.014	0.015	0.016	0.016	0.017	0.017	0.018	0.019	0.022	0.025	0.03	0.036	0.043	0.053	0.069	0.092	0.108	0.109	0.11	0.11
9	**$C_{m\alpha }$**	0.0025	0.0025	0.0025	0.0025	0.0025	0.0025	0.0025	0.0025	0.0025	0.0025	0.0025	0.0025	0.0025	0.0025	0.0025	0.0025	0.0025	0.0025	0.0025	0.0025	0.0025	0.0025	0.0025	0.0025
10	**$C_{m0}$**	0.002	0.002	0.001	0.001	0	**−0.00013**	−0	−0.001	−0.001	−0.002	−0.003	−0.004	−0.006	−0.008	−0.01	−0.01	−0.01	−0.02	−0.02	−0.02	−0.021	−0.022	−0.021	−0.022

## References

[1] Hundley R.O., Gritton E.C. (1994). Future Technology-Driven Revolutions in Military Operations.

[2] Kellogg J., Bovais C., Foch R., McFarlane H., Sullivan C., Dahlburg J., Gardner J., Ramamurti R., Gordon-Spears D., Hartley R. (2002). The NRL micro tactical expendable (MITE) air vehicle. Aeronaut. J..

[3] Grasmeyer J., Keennon M. Development of the black widow micro air vehicle. Proceedings of the 39th Aerospace Sciences Meeting and Exhibit.

[4] Bronz M., Condomines J.P., Hattenberger G. (2013). Development of an 18 cm Micro Air Vehicle: Quark.

[5] Spoerry M.T., Wong K. (2001). Design and development of a micro air vehicle (μav) concept: Project Bidule. Proceedings of the 9th Annual International Aerospace Congress, School of Aerospace, Mechanical and Mechatronic Engineering.

[6] Bronz M., Hattenberger G., Moschetta J.M. (2013). Development of a long endurance mini-uav: Eternity. Int. J. Micro Air Veh..

[7] Petricca L., Ohlckers P., Grinde C. (2011). Micro-and nano-air vehicles: State of the art. Int. J. Aerosp. Eng..

[8] McLain T.W., Beard R.W., Barber D.B., Knoebel N.B. (2007). An Overview of MAV Research at Brigham Young University.

[9] Cai G., Dias J., Seneviratne L. (2014). A survey of small-scale unmanned aerial vehicles: Recent advances and future development trends. Unmanned Syst..

[10] Embacher M., Fasel H.F. (2014). Direct numerical simulations of laminar separation bubbles: investigation of absolute instability and active flow control of transition to turbulence. J. Fluid Mech..

[11] McGhee R.J. (1988). Experimental Results for the Eppler 387 Airfoil at Low Reynolds Numbers in the Langley Low-Turbulence Pressure Tunnel.

[12] Bastedo W.G., Mueller T.J. (1986). Spanwise variation of laminar separation bubbles on wings at low Reynolds number. J. Aircr..

[13] Pelletier A., Mueller T.J. (2000). Low Reynolds number aerodynamics of low-aspect-ratio, thin/flat/cambered-plate wings. J. Aircr..

[14] Mueller T.J., DeLaurier J.D. (2003). Aerodynamics of small vehicles. Annu. Rev. Fluid Mech..

[15] McGranahan B., Selig M. Surface oil flow measurements on several airfoils at low Reynolds numbers. Proceedings of the 21st AIAA Applied Aerodynamics Conference.

[16] Torres G.E., Mueller T.J. (2004). Low aspect ratio aerodynamics at low Reynolds numbers. AIAA J..

[17] Cosyn P., Vierendeels J. (2007). Design of fixed wing micro air vehicles. Aeronaut. J..

[18] Spedding G., McArthur J. (2010). Span efficiencies of wings at low Reynolds numbers. J. Aircr..

[19] Ananda G., Sukumar P., Selig M.S. (2015). Measured aerodynamic characteristics of wings at low Reynolds numbers. Aerosp. Sci. Technol..

[20] Shams T.A., Shah S.I.A., Ahmad M.A. Study of Low Reynolds Number Aerodynamics for Low Aspect Ratio MAV Wing. Proceedings of the 2018 IEEE 21st International Multi-Topic Conference (INMIC).

[21] Stevens B.L., Lewis F.L., Johnson E.N. (2015). Aircraft Control and Simulation: Dynamics, Controls Design, and Autonomous Systems.

[22] Roskam J. (1998). Airplane Flight Dynamics and Automatic Flight Controls.

[23] Raymer D. (2012). Aircraft Design: A Conceptual Approach.

[24] McLain T.W., Beard R.W. Unmanned air vehicle testbed for cooperative control experiments. Proceedings of the 2004 American Control Conference.

[25] Drela M. (1989). XFOIL: An analysis and design system for low Reynolds number airfoils. Low Reynolds Number Aerodynamics.

[26] De Tavernier D., Baldacchino D., Ferreira C. (2018). An integral boundary layer engineering model for vortex generators implemented in XFOIL. Wind. Energy.

[27] Coder J.G., Maughmer M.D. (2015). Numerical validation of the Squire–Young formula for profile-drag prediction. J. Aircr..

[28] Morgado J., Vizinho R., Silvestre M., Páscoa J. (2016). XFOIL vs CFD performance predictions for high lift low Reynolds number airfoils. Aerosp. Sci. Technol..

[29] Selig M.S., Guglielmo J.J. (1997). High-lift low Reynolds number airfoil design. J. Aircr..

[30] Maughmer M.D., Coder J.G. (2010). Comparisons of Theoretical Methods for Predicting Airfoil Aerodynamic Characteristics.

[31] Anderson J.D. (1999). Aircraft Performance and Design.

[32] Nelson R.C. (1998). Flight Stability and Automatic Control.

[33] Song L., Yang H., Zhang Y., Zhang H., Huang J. (2014). Dihedral influence on lateral–directional dynamic stability on large aspect ratio tailless flying wing aircraft. Chin. J. Aeronaut..

[34] Huyssen R.J. (2020). Tailless Aircraft. US Patent.

[35] Lambert W.B., Stanek M.J., Gurka R., Hackett E.E. (2019). Leading-edge vortices over swept-back wings with varying sweep geometries. R. Soc. Open Sci..

[36] Barlow J.B., Rae W.H., Pope A. (2015). Low speed wind tunnel testing. INCAS Bull..

[37] White F.M. (1999). Fluid Mechanics.

[38] Helmbold H. (1942). Der unverwundene ellipsenflugel als tragende flanche. Jahrbuch.

[39] Kuchemann D. (1965). The Aerodynamic Design of Aircraft. Prog. Aeronaut. Sci..

[40] Harper C.W., Maki R.L. (1964). A Review of the Stall Characteristics of Swept Wings.

[41] Visbal M.R., Garmann D.J. (2019). Effect of sweep on dynamic stall of a pitching finite-aspect-ratio wing. AIAA J..

[42] Phillips E., Wygnanski I. Using Sweeping Jets on Swept Wings. Proceedings of the FLUCOME.

[43] Prisacariu V. (2018). Analysis of UAVS flight characteristics. Rev. Air Force Acad..

[44] Murua J., Palacios R., Graham J.M.R. (2012). Applications of the unsteady vortex-lattice method in aircraft aeroelasticity and flight dynamics. Prog. Aerosp. Sci..

[45] Drela M., Youngren H. (2019). XFOIL 6.94 User Guide, 2001.

[46] Jana S., Kandath H., Shewale M., Bhat M.S. (2020). Effect of propeller-induced flow on the performance of biplane micro air vehicle dynamics. Proc. Inst. Mech. Eng. Part G J. Aerosp. Eng..

[47] Gamble B.J., Reeder M.F. (2009). Experimental analysis of propeller-wing interactions for a micro air vehicle. J. Aircr..

[48] Durai A. (2014). Experimental investigation of lift and drag characteristics of a typical MAV under propeller induced flow. Int. J. Micro Air Veh..

[49] Aminaei H., Dehghan Manshadi M., Mostofizadeh A.R. (2019). Experimental investigation of propeller slipstream effects on the wing aerodynamics and boundary layer treatment at low Reynolds number. Proc. Inst. Mech. Eng. Part G J. Aerosp. Eng..

[50] Katz J., Feistel T.W. (1987). Propeller swirl effect on single-engine general-aviation aircraft stall-spin tendencies. J. Aircr..

[51] Sinha S.K., Turner K.E. (2011). Natural frequencies of a pre-twisted blade in a centrifugal force field. J. Sound Vib..

